# The Fanconi Anemia Group C Protein Interacts with Uncoordinated 5A and Delays Apoptosis

**DOI:** 10.1371/journal.pone.0092811

**Published:** 2014-03-27

**Authors:** FengFei Huang, Manel Ben Aissa, Audrey Magron, Caroline C. Huard, Chantal Godin, Georges Lévesque, Madeleine Carreau

**Affiliations:** 1 Department of Pediatrics, Centre Hospitalier Universitaire de Québec, Québec, Québec, Canada; 2 Department of Psychiatry and Neurosciences, Université Laval, Cité Universitaire, Québec, Canada; Oregon Health and Science University, United States of America

## Abstract

The Fanconi anemia group C protein (FANCC) is one of the several proteins that comprise the Fanconi anemia (FA) network involved in genomic surveillance. FANCC is mainly cytoplasmic and has many functions, including apoptosis suppression through caspase-mediated proteolytic processing. Here, we examined the role of FANCC proteolytic fragments by identifying their binding partners. We performed a yeast two-hybrid screen with caspase-mediated FANCC cleavage products and identified the dependence receptor uncoordinated-5A (UNC5A) protein. Here, we show that FANCC physically interacts with UNC5A, a pro-apoptotic dependence receptor. FANCC interaction occurs through the UNC5A intracellular domain, specifically via its death domain. FANCC modulates cell sensitivity to UNC5A-mediated apoptosis; we observed reduced UNC5A-mediated apoptosis in the presence of FANCC and increased apoptosis in FANCC-depleted cells. Our results show that FANCC interferes with UNC5A's functions in apoptosis and suggest that FANCC may participate in developmental processes through association with the dependence receptor UNC5A.

## Introduction

The Fanconi anemia group C protein (FANCC) is a multifunctional protein, with roles in several cellular processes, such as DNA damage signaling, redox regulation, transcriptional regulation, and apoptosis [1]. Mutations in the *FANCC* gene lead to Fanconi anemia, a genetic disease characterized by a progressive depletion of bone marrow cells [2], [3]. This disease is also associated with various congenital malformations and an increased risk of malignancies [4]. There are 16 FA disease-associated genes that form an entity known as the FA pathway that enacts a global response to DNA crosslink damage [5]–[7]. FANCC is the cytoplasmic component of the FA pathway, and in association with the Fanconi anemia group E (FANCE) protein, translocates to the nucleus in response to crosslink damage [8]–[10]. Nuclear FANCC associates with other components of the FA pathway to compose the FA core complex. Besides this association with FA core complex proteins, FANCC binds several proteins involved in various cellular functions, such as oxygen radical metabolism, signal transduction, transcription, and apoptosis [1], [11]. FANCC has mostly been studied in the context of cell survival and death signaling. For instance, FANCC-deficient cells show increased apoptosis in response to inhibitory cytokines, serum deprivation, apoptosis inducers, DNA crosslink damage, and reactive oxygen species [12]–[14]. FANCC over-expression attenuated apoptosis and induced a survival response in non-FA cells, thus, FANCC is considered a survival or anti-apoptotic protein [12], [14]–[20]. We have previously shown that in response to apoptosis, FANCC undergoes caspase-mediated proteolytic processing, leading to the generation of cleaved protein fragments [15]. Cleaved FANCC is not able to suppress apoptosis, but a non-cleavable form of FANCC further delays its onset [15]. Currently, little is known about the molecular events leading to FANCC cleavage and its impact on downstream cellular signaling. To further characterize the cellular functions of FANCC, we performed yeast two-hybrid screens using FANCC cleavage products to identify protein interactors. Among the candidates obtained, one candidate coded for the dependence receptor uncoordinated-5 A (UNC5A).

UNC5A is a member of the netrin-1 transmembrane receptor family that is comprised of four homologs, namely UNC5A, UNC5B, UNC5C, and UNC5D, also called UNC5H1, UNC5H2, UNC5H3, and UNC5H4. The UNC5 receptors are single-pass type I transmembrane proteins that contain two immunoglobulin repeats followed by two thrombospondin type-I repeats in the extracellular domain [21]. The intracellular region of UNC5A contains a PEST zona occludens-1 homology domain (ZU-5), a deleted in colorectal cancer (DCC)-binding domain, and a death domain (DD). UNC5 proteins have been proposed to function as proapoptotic “dependence receptors” that trigger apoptosis in the absence of their ligand [22]. UNC5-mediated apoptosis occurs via the ZU5 domains or DDs [23], [24]. Expression studies in mice have shown that UNC5 receptors are expressed in early eye development, mammary bud formation, vascularization, and limb development [25]. In addition, loss of UNC5 gene expression is associated with various cancers and tumor aggressiveness, supporting the hypothesis that UNC5 proteins act as tumor suppressors [26].

Here, we show a direct interaction between FANCC and UNC5A cytoplasmic death domain. We also show that FANCC delays UNC5A-mediated apoptosis.

## Materials and Methods

### Plasmids and DNA constructs

The N-terminus of FANCC, which spans from nucleotides 256 to 1175 and encompasses amino acids from the start codon to the cleavage site [15], was cloned into the pGBKT7 and pGADT7 yeast vectors (Clontech Laboratories Inc., Mountain View, CA) by fusion to the Gal4-DNA binding or DNA-activating domain, and into the pEGFP plasmids (pGBKFANCC^1-306^, pGADFANCC^1-306^, pEGFPFANCC^1-306^). Similarly, the C-terminus of FANCC, spanning from nucleotides 1176 to 1929 and corresponding to the cleaved FANCC C-terminus fragment amino acids 307 to 558, was cloned into the pGBKT7 and pGADT7 yeast vectors (pGBKFANCC^307-558^ and pGADFANCC^307-558^) and the pEGFP plasmid (pEGFPFANCC^307-558^), as previously described [15]. The pGADT7-FANCC^307-558-L554P^ and pGBKT7-FANCC^307-558-L554P^ constructs were obtained by site-directed mutagenesis using the FANCC^307-558^ coding plasmids and the Quikchange II site-directed mutagenesis kit according to the manufacturer's protocol (Agilent Technologies, Mississauga, ON). Other FA gene constructs have been described previously [27]. The Myc-tagged rat UNC5A plasmid (pSecrUnc5A) was generously provided by Dr. Tessier-Lavigne. Upon sequence verification of this plasmid, we noticed that part of the 5′ end (118 bp) and 1000 bp of the 3′ end of the full length Unc5A cDNA was missing. We therefore cloned the full-length human UNC5A and the intracellular domain of the human UNC5A spanning from nucleotides 985 to 2529 of the open reading frame (corresponding to amino acids 329 to 842) by RT-PCR into pCMVzeo vectors in frame with an HA-tag (pCMVzeoUNC5A^ICD^). Subsequently, UNC5A and UNC5A^329-842^ (UNC5A^ICD^) were subcloned into pGBKT7 and pGADT7 yeast vectors and pCDNAHisXpres and pCDH-CMV-MCS lentiviral vectors. UNC5A with a deleted DD (UNC5A^ΔDD^) was cloned into pGBKT7 and pGADT7 yeast vectors. Lentiviral vectors TRCN0000083368, TRCN0000083369, TRCN0000083370, TRCN0000083371 and TRCN0000083372 coding for shRNA against FANCC; V2LHS-74072, V2LHS-74073, V3LHS-321475, V3LHS-321477, V3LHS-321480 coding for shRNA against FANCE; V2LHS-16512, V2LHS-16513, V2LHS-304038, V2LHS-304039, V2LHS-304040 coding for shRNA against UNC5A were obtained from Thermo Scientific (ThermoFisher Scientific, Mississauga, ON).

### Antibodies

The following antibodies were used: previously described 8F3 anti-FANCC [15], a gift from Dr. M. Hoatlin (OHSU), and Novus Biologicals (Littleton, CO); anti-FANCA (Santa Cruz Biotechnologies, Santa Cruz, CA), anti-UNC5A (Sigma-Aldrich, St. Louis, MO); anti-HA (12CA5, Roche Diagnostics, Indianapolis, IN); anti-cMyc (9E10, Santa Cruz Biotechnologies, Santa Cruz, CA); anti-GFP (clone B2; Santa Cruz Biotechnologies); anti-cleaved-caspase-3 (Cell Signaling Technologies, Danvers, MA), anti-mouse and anti-rabbit (Santa Cruz Biotechnologies); and donkey anti-rabbit Alexa Fluor 488 or 555 and anti-mouse Alexa Fluor 555 or 488 (Invitrogen, Burlington, ON). F-actin was labeled with Alexa Fluor 546 phalloidin (Life Technologies, Burlington, ON).

### Yeast two-hybrid screens and analyses

Yeast two-hybrid screens and analyses were performed using the MATCHMAKER Two-Hybrid System 3 according to the manufacturer's instructions (Clontech). The FANCC N- and C-terminal fragment (pGBKFANCC^1-306^ and pGBKFANCC^307-558^, respectively) bait constructs were tested for self-activation in the yeast two-hybrid assay prior to use with library screens and were found to be free of autonomous Gal-4 activation. Yeast two-hybrid screens were performed according to the manufacturer's protocol (Clontech) using the MATCHMAKER Two Hybrid System 3, where the bait FANCC constructs were transformed into the AH109 *Saccharomyces cerevisiae* yeast strain containing the nutritional reporter genes *ADE2* and *HIS3*. The transformed AH109 yeasts were mated with the Y187 *S. cerevisiae* strain, which was pre-transformed with cDNA libraries obtained from either HeLa cells or human fetal brain (Clontech). Yeast diploids were selected by plating on dropout medium lacking tryptophan and leucine (-TL), and by detecting protein-protein interactions on dropout medium lacking tryptophan, leucine, histidine, and adenine (-TLHA). Three different screens were performed using each pGBKT7-FANCC^1-306^ and pGBKT7-FANCC^307-558^ construct as the bait.

For yeast two-hybrid analyses using FA proteins, all FA genes were subcloned into yeast two-hybrid vectors, as previously described [27]. Constructs were co-transformed into the AH109 *S. cerevisiae* strain (Clontech) and selected for growth and reporter gene activation. Positive controls included pGBKT7-p53 with the pGADT7-T antigen and pGBKT7-FANCF with pGADT7-FANCG. Negative controls were empty pGBKT7 or pGADT7 vectors in combination with the corresponding FA gene-coding plasmid. Each construct was sequenced and tested for autonomous Gal-4 activation, and FANCG and FANCL showed self-activation when cloned into pGBKT7 [27], [28]. Each experiment was performed at least three times in triplicate and with each gene cloned into either the pGBKT7 or pGADT7 vectors.

### Cells, cultures, and transfection

HEK293T and HeLa cells were grown at 37°C, 5% CO2 in Dulbecco's modified Eagle's medium (DMEM) supplemented with 10% fetal calf serum (FCS). SH-SY5Y cells were grown at 37°C, 5% CO2 in a 1∶1 mixture of DMEM and Ham's F12 nutrient mixture (HyClone, ThermoFisher Scientific) and 10% FCS. Cells were transfected using the calcium-phosphate method or Lipofectamine 2000 (Invitrogen). For cellular depletion, SH-SY5Y cells were transduced with lentiviral particles coding for a mixture of 5 different shRNA against FANCC or UNC5A or scrambled sequences. Lentiviral particles were produced using the four-plasmid expression system containing pRSV-Rev, pMDLg/pRRE, pMD2.G and the different expression vectors as previously described [11]. Following transduction, cells were selected and maintained in media containing puromycin (2.0 ug/ml, Life technologies, Burlington, ON). For induction of neurite outgrowth, SH-SY5Y cells were plated in collagen-coated 6-well plates at a density of 2 X 10^4^ cells/cm^2^ and were deprived of serum (0.5% FCS) for 24 to 48 hours and treated with retinoic acid (10 μM, Sigma-Aldrich) or DMSO for up to 6 days or treated with staurosporin (25 nM, Sigma-Aldrich) for 72 hours. Cells were treated with recombinant human netrin-1 (500 ng/ml; R&D systems, Minneapolis, MN) for 4 hours prior to immunofluorescence staining. For neurite length estimation, cells were visualized using a Nikon E300 inverted microscope (Nikon Canada, Mississauga, ON) at 40X magnification. At least 200 cells from microscopic fields selected at random were counted in each sample.

### Immunoblot analysis and immunoprecipitations

For immunoblot analysis, total cell lysates were prepared in sodium dodecyl sulfate (SDS)-loading buffer (50 mM Tris-HCl, 2% 2-mercaptoethanol, 2% SDS). Samples were sonicated and/or boiled and subjected to electrophoresis on 10% or 12% SDS-polyacrylamide gels. Proteins were electrotransferred onto a PVDF membrane (Amersham) and probed with antibodies, as indicated in each figure. For immunoprecipitation (IP), 5×10^6^ to 1×10^7^ cells were harvested, washed in phosphate-buffered saline (PBS), and resuspended in lysis buffer (50 mM Tris-HCl, 150 mM NaCl, 1% Triton X-100, and complete proteases inhibitors [Roche Diagnostics]). Lysates were cleared by centrifugation and mixed with 1 to 2 μg of the precipitating antibody, as indicated in the figure. Antibody-antigen complexes were pulled down with protein A-agarose beads (Calbiochem, San Diego, CA) for over-expressed proteins or Dynabeads (Invitrogen) for endogenous protein complexes. Immunoprecipitates were resolved by SDS-polyacrylamide gel electrophoresis on 10% or 12% polyacrylamide gels and subjected to Western blotting with specific antibodies, as indicated in each figure. Control IPs were performed using either mouse or rabbit IgGs, as indicated in the figures.

### Immunofluorescence microscopy

For localization of FANCC and UNC5A, immunofluorescence microscopy was performed as follows. SH-SY5Y cells were grown in the appropriate culture condition on poly-L-lysine coated coverslips (12-mm diameter) prior to treatment. Cells were fixed with either 4% paraformaldehyde in PBS for 20 minutes at room temperature or methanol/acetone (3∶7) for 20 minutes at -20°C, followed by permeabilization for 15 minutes at room temperature with 0.3% Triton X-100 in PBS or 1 hour with 0.1% saponin with 2% BSA in PBS. Fixed cells were incubated with specific primary antibodies as indicated in the figures, followed by secondary antibodies (goat anti-mouse Alexa Fluor 488, goat anti-rabbit Alexa Fluor 546, or donkey anti-rabbit Alexa Fluor 555) at the appropriate dilution in PBS with 10% horse serum or with 0.1% saponin with 2% BSA. Following labeling with primary and secondary antibodies, cells were washed three times with PBS. Images were acquired using a Nikon E800 fluorescent microscope equipped with a C1 confocal system (Nikon Canada, Mississauga, ON).

### Apoptosis assays

Apoptosis was induced in SH-SY5Y cells transfected with the appropriate DNA constructs by 4-hour staurosporin treatment (1 μM; Roche Diagnostics). Following apoptosis induction, caspase-3 activation was assessed with a Caspase-3, Active Form, mAb Apoptosis Kit according to manufacturer's instructions (BD Biosciences, Mississauga, ON). Caspase-3-positive cells were analyzed by confocal microscopy and manually scored for both GFP-positive (transfected cells) and caspase-3-positive cells, or analyzed by flow cytometry (BD SORP LSR II; BD Biosciences) by gating for GFP- and caspase-3-positive cells.

### Statistical analyses

Data were expressed as means ± standard errors of the means (SEMs). Statistical analyses were performed using the GraphPad Prism software (version 5.0b; GraphPad Software Inc., San Diego, CA), and the paired and unpaired two-tailed Student's t-tests were used to compare the means. P values less than 0.05 were considered significant.

## Results

### Identifying novel partners of FANCC

To gain further insight into FANCC's role in apoptosis we performed yeast-2-hybrid screens with FANCC's caspase-mediated proteolytic fragments. We used both N-terminal (FANCC^1-306^) and C-terminal (FANCC^307-558^) regions of FANCC corresponding to cleavage products as baits for yeast two-hybrid analysis. Two separate and independent screens were performed using a fetal brain cDNA library as prey. Most protein candidates were obtained with screens using FANCC^1-306^ as bait (Table 1). Among the positive yeast colonies that were obtained with FANCC^1-306^, two independent and strong positive candidates encoded the C-terminal portion of the UNC5A protein (amino acids 729 to 842). Of the candidates obtained, one had been previously identified by another group, notably, the FANCG-interacting protein peroxiredoxin-3 (clone 5.6) [29], whereas the C-terminal-binding protein 1 (clone 11.1) has been previously published by us [11]. All clones were retested in yeast two-hybrid assays against FANCC^1-306^ or the empty bait vector (Table 2). Eight of the eleven clones that were retested in yeasts assays, including UNC5A, showed positive interaction with FANCC^1-306^, whereas three candidates did not show interaction with FANCC^1-306^ when plated on selective media (-TLHA).

**Table 1:** Candidate clones obtained from Yeast-2-hybrid screens.

Clone number	Accession number	Gene	E-value	ID (%)
1.1	NM_001243743.1	FANCC	0.0	62
2.1	BC157824.1	UNC5A	0.0	99
3.4	BC157824.1	UNC5A	0.0	99
4.1	NM_000721.3	CACNAE1	0.0	100
5.6	AK313169.1	PRDX3	0.0	99
6.1	NM_007029.3	STMN2	0.0	99
6.4	NM_007029.3	STMN2	0.0	99
7.4	NM_007029.3	STMN2	0.0	99
8.1	AC009754.10	RP11-519C12	1e-114	76
11.1	NM_001328.2	CtBP1	0.0	99
12.2	AY207372.1	CCN1	4e-154	77

% ID: percent gene identity.

**Table 2:** Yeast-2-hybrid assays between FANCC^1-306^ and the identified clones.

	pGBKFANCC^1-306^		pGBKT7	
pACT2 clones	-TL	-TLHA	-TL	-TLHA
1.1 FANCC	+	-	+	-
2.1 UNC5A	+	+	+	-
3.4 UNC5A	+	+	+	-
4.1 CACNAE1	+	-	+	-
5.6 PRDX3	+	+	+	-
6.1 STMN2	+	+	+	-
6.4 STMN2	+	+	+	-
7.4 STMN2	+	+	+	-
8.1 RP11-519C12	+	-	+	-
11.1 CtBP1	+	+	+	-
12.2 CCN1	+	+	+	-

+ indicates growth on selective media.

### FANCC interacts with UNC5A

**Figure 1:**
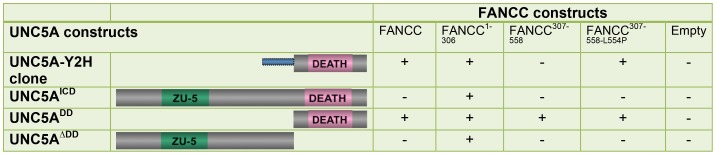
FANCC directly interacts with UNC5A in yeasts. Yeast two-hybrid assays were performed with various UNC5A constructs that included the Y2H clone coding for the C-terminal death domain (DEATH) and a part of intron 14 (shown in blue), UNC5A intracellular domain (UNC5A^ICD^), the UNC5A C-terminus (UNC5A^DD^) and the death-domain deletion mutant (UNC5A^ΔDD^). The yeast strain AH109 was co-transformed with UNC5A constructs along with FANCC constructs as indicated. The plus sign (+) indicate a positive interaction. Negative controls were performed using empty vectors (Empty), and positive controls were performed using pGBKT7/p53 and pGADT7/SV40 T-antigen from Clontech (data not shown). Yeast two-hybrid assays were performed at least three times (independent transformation) with 3 to 4 clones each.

We selected the UNC5A candidate and subcloned it into pGBKT7 and pGADT7 yeast vectors. These we subsequently tested in yeast two-hybrid assays against FANCC^1-306^, FANCC^307-558^ and the full-length FANCC protein. Results showed that the UNC5A clone corresponding to the C-terminal region directly interacts with FANCC^1-306^, as well as with full-length FANCC but not with FANCC^307-558^ (Figure 1). Sequencing analysis revealed that the UNC5A clone contained intronic sequences (129 bp upstream the exon 15 splicing site) corresponding to parts of intron 14 of the *UNC5A* gene (Figure 1, shown in blue). Therefore, we generated yeast vectors containing cDNA corresponding to the intracellular domain of the UNC5A protein (UNC5A^ICD^), to the death domain deletion mutant (UNC5A^ΔDD^) and to the C-terminus part containing the death domain (UNC5A^DD^).

As shown by yeast two-hybrid analysis, the intracellular domain of UNC5A (UNC5A^ICD^) showed positive interaction with FANCC^1-306^ but not full-length FANCC or FANCC C-terminus domain (FANCC^307-558^). However, results show that the UNC5A C-terminus containing the death domain interacts directly with full-length FANCC and both FANCC fragments. In addition, FANCC^1-306^ but not full length FANCC or FANCC^307-558^ showed positive interaction with UNC5A lacking the death domain (UNC5A^ΔDD^). All UNC5A and FANCC constructs were found devoid of self-activation as shown by negative growth with empty vectors on stringent nutritional selection (Figure 1). These results suggest that FANCC^1-306^ binds UNC5A via more than one region, whereas FANCC^307-558^ and full-length FANCC interacts with UNC5A death domain. Next, to determine whether a FA-causing mutation of FANCC impacted its ability to interact with UNC5A, we generated a FANCC C-terminus construct harboring the L554P mutation (FANCC^307-558-L554P^) found in patients with FA. Results showed that the interaction between the UNC5A C-terminus and FANCC^307-558-L554P^ still occurred in yeasts. These results imply that a mutated FANCC protein may conserve interaction with UNC5A.

### FANCC immunoprecipitates with UNC5A

**Figure 2:**
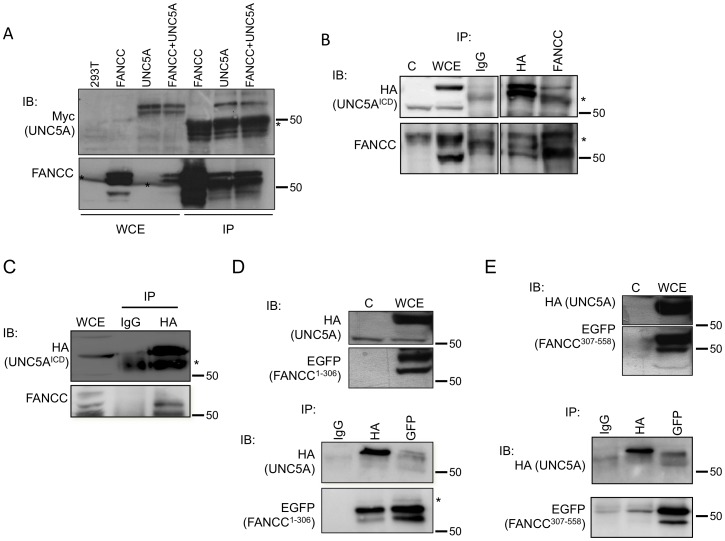
FANCC interacts with UNC5A. Immunoprecipitations (IPs) were performed in HEK293T cells transiently transfected with Myc-tagged rUNC5A (A), HA-tagged UNC5A^ICD^ (B–C), with (A–B) or without (C) full-length FANCC. In D and E, HEK293T cells were cotransfected with HA-tagged UNC5A^ICD^ and EGFP-tagged FANCC^1-306^ (D) or EGFP-tagged FANCC^307-558)^ (E). IPs were performed using anti-FANCC, anti-Myc, anti-HA, anti-GFP or control mouse serum (IgG). Western immunoblotting (IB) was performed with the indicated antibodies. WCE: whole cell extract from transfected cells, C: control untransfected cells. * indicates non-specific or IgG bands. Numbers indicate molecular weight.

To determine whether FANCC interaction with UNC5A occurs in cells, we first performed immunoprecipitation with cell extracts overexpressing UNC5A and FANCC. To do so, we obtained the rat UNC5A cDNA coding vector pSecUNC5A (generously provided by Dr Tessier-Lavigne) and performed coimmunoprecipitation experiments (Figure 2A). Although FANCC coimmunoprecipitated with the rat UNC5A protein, sequencing analysis of this plasmid revealed a partial open-reading frame where 118 bp were missing from the 5′ end and 1000 bp missing from the 3′ end, which encodes the death domain. However, these results supports data obtained in yeasts assays showing that UNC5A interaction with FANCC occurs through different UNC5A protein domains. Next, we amplified the human cDNA of the intracellular domain (ICD) of UNC5A and cloned it into mammalian expression vectors. We then performed co-immunoprecipitation studies in cells expressing the HA-tagged UNC5A^ICD^ and FANCC. Immunoprecipitation was performed with either anti-HA or anti-FANCC antibodies. Immunoblot analyses revealed that full-length FANCC co-immunoprecipitates with UNC5A^ICD^ (Figure 2B). Immunoprecipitation performed with HA-tagged UNC5A^ICD^ and endogenous FANCC confirms results obtained with overexpressed proteins (Figure 2C).

Next, we performed co-immunoprecipitation studies in cells expressing EGFP-tagged FANCC^1-306^ or FANCC^307-558^ with UNC5A^ICD^ by using anti-GFP and anti-HA (HA-UNC5A^ICD^) antibodies. Western blot analyses showed that the immunoprecipitates contained UNC5A^ICD^ and the FANCC N-terminus (Figure 2D, lower panel) or the C-terminus regions (Figure 2E, lower panel). Together, these results and data obtained in yeasts suggest that FANCC protein fragments interact with different UNC5A protein domains.

**Figure 3:**
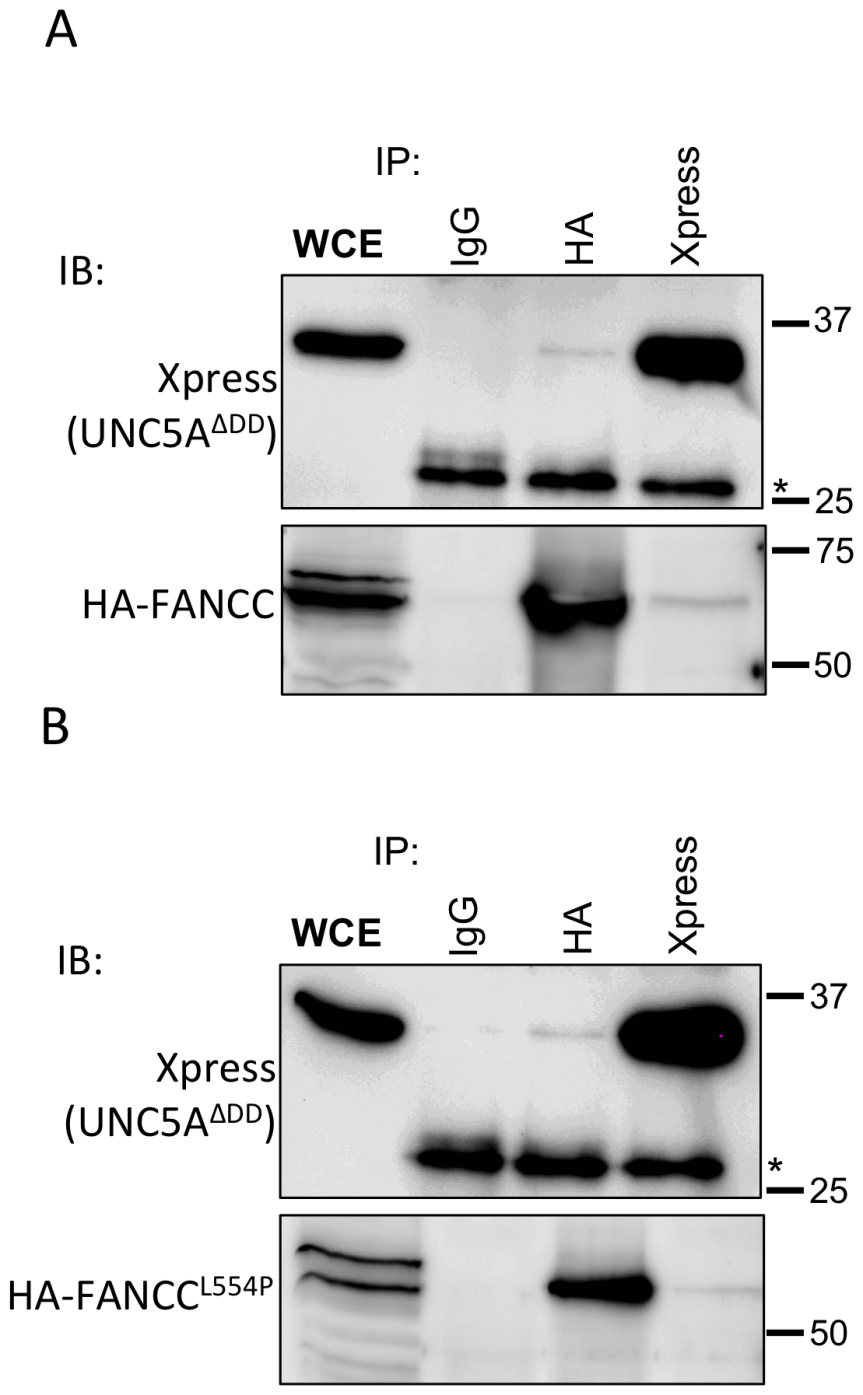
FANCC interacts with UNC5A^ΔDD^. Immunoprecipitations (IPs) were performed in HEK293T cells transiently transfected with Xpress-tagged death-domain deletion mutant UNC5A (UNC5A^ΔDD^) and HA-tagged FANCC (A) or in B HA-tagged FANCC harboring the L554P mutation (HA-FANCC^L554P^). IPs were performed using anti-Xpress, anti-HA or control mouse serum (IgG). Western immunoblotting (IB) was performed with the indicated antibodies. WCE: whole cell extract from transfected cells. * indicates non-specific or IgG bands. Numbers indicate molecular weight

**Figure 4:**
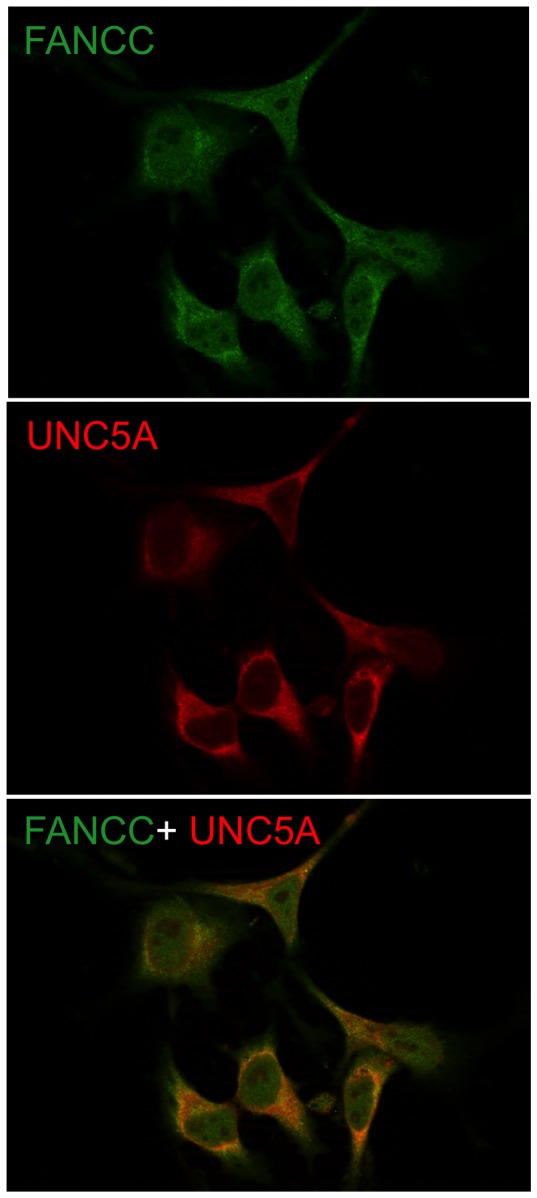
FANCC co-localizes with UNC5A in the cytoplasm. Representative immunofluorescence experiment performed in HEK293T cells double-stained with anti-UNC5A (red) and anti-FANCC (green) antibodies. Cells were visualized via confocal fluorescence microscopy using a Nikon E800 microscope equipped with a C1 confocal system at 100X magnification.

To determine whether FANCC interaction with UNC5A in cells requires the UNC5A death domain, we performed immunoprecipitation analysis using protein extracts from cells expressing full-length FANCC and a UNC5A death domain deletion mutant (UNC5A^ΔDD^). Immunoprecipitation were performed with antibodies against the Xpress epitope tag of UNC5A or the HA epitope tag of FANCC. Although results show that FANCC still co-immunoprecipitated with UNC5A^ΔDD^ only a faint FANCC protein band is detected in the Xpress-mediated IP lane or a faint UNC5A band in the HA-mediated IP lane (Figure 3A). Because no protein is detected in the control IgG immunoprecipitates, results suggest that a week interaction occurs between FANCC and UNC5A^ΔDD^. These results also imply that the UNC5A death domain may be required for strong FANCC binding in cells or that a protein present in cells but absent in yeasts mediates FANCC interaction with UNC5A^ΔDD^. Next, to determine whether full-length FANCC harboring the L554P mutation retained the interaction with UNC5A as in yeasts, we performed immunprecipitation analyses using a HA-tagged full-length FANCC^L554P^ mutant. Results show that a week interaction between FANCC^L554P^ and UNC5A^ΔDD^ occurred (Figure 3B). Together, these results indicate that FANCC interacts with UNC5A in cells and that the L554P mutation in FANCC does not disrupt this interaction. These results also indicate that the UNC5A death domain is required for strong FANCC binding. As expected, immunofluorescence analysis indicated that endogenous UNC5A is found in the cytoplasm and colocalizes with cytoplasmic FANCC (Figure 4). Collectively, these results indicate that FANCC interacts with UNC5A.

### FANCC prevents UNC5A-mediated apoptosis

**Figure 5:**
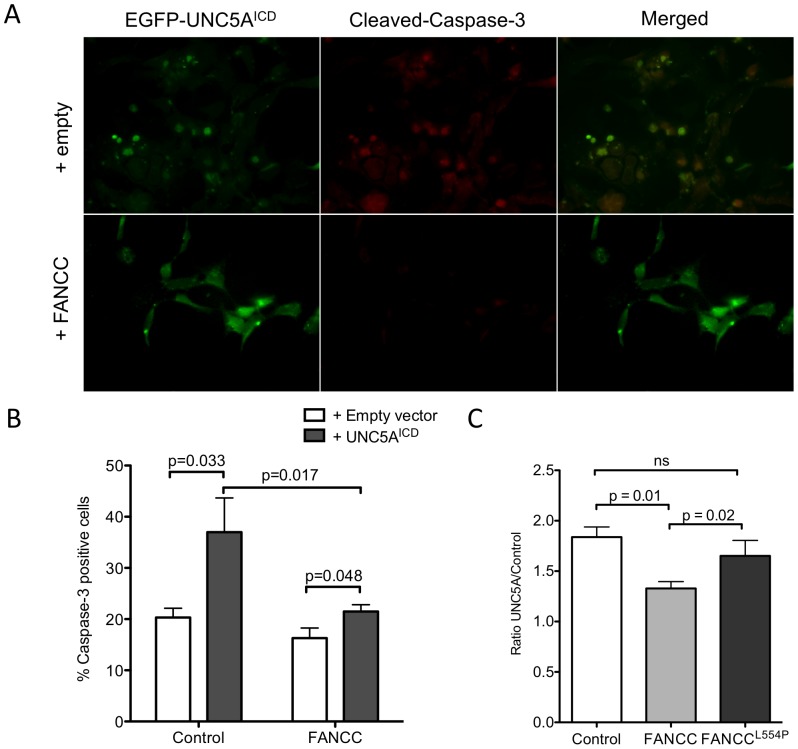
FANCC interferes with UNC5A-mediated apoptosis. HeLa cells were transiently transfected with EGFP-UNC5A^ICD^ with or without full-length FANCC or empty vectors. At 48 hours after transfection, cells were fixed and labeled with anti-cleaved-caspase-3 antibodies, and visualized by fluorescence microscopy using a Nikon E800 microscope equipped with a C1 confocal system at 60X magnification. (A) Representative immunofluorescence experiment showing EGFP-UNC5A^ICD^ transfected cells (green) and cleaved-caspase-3 positive cells (red). (B) Data are expressed as the mean percent ± standard error of the mean (SEM) of cleaved-caspase-3-positive cells out of EGFP-positive cells from 3 separate experiments. (C) Data are expressed as ratio of caspase-3-positive cells obtained from EGFP-positive UNC5A^ICD^ cells compared to cells transfected with empty vectors.

**Figure 6:**
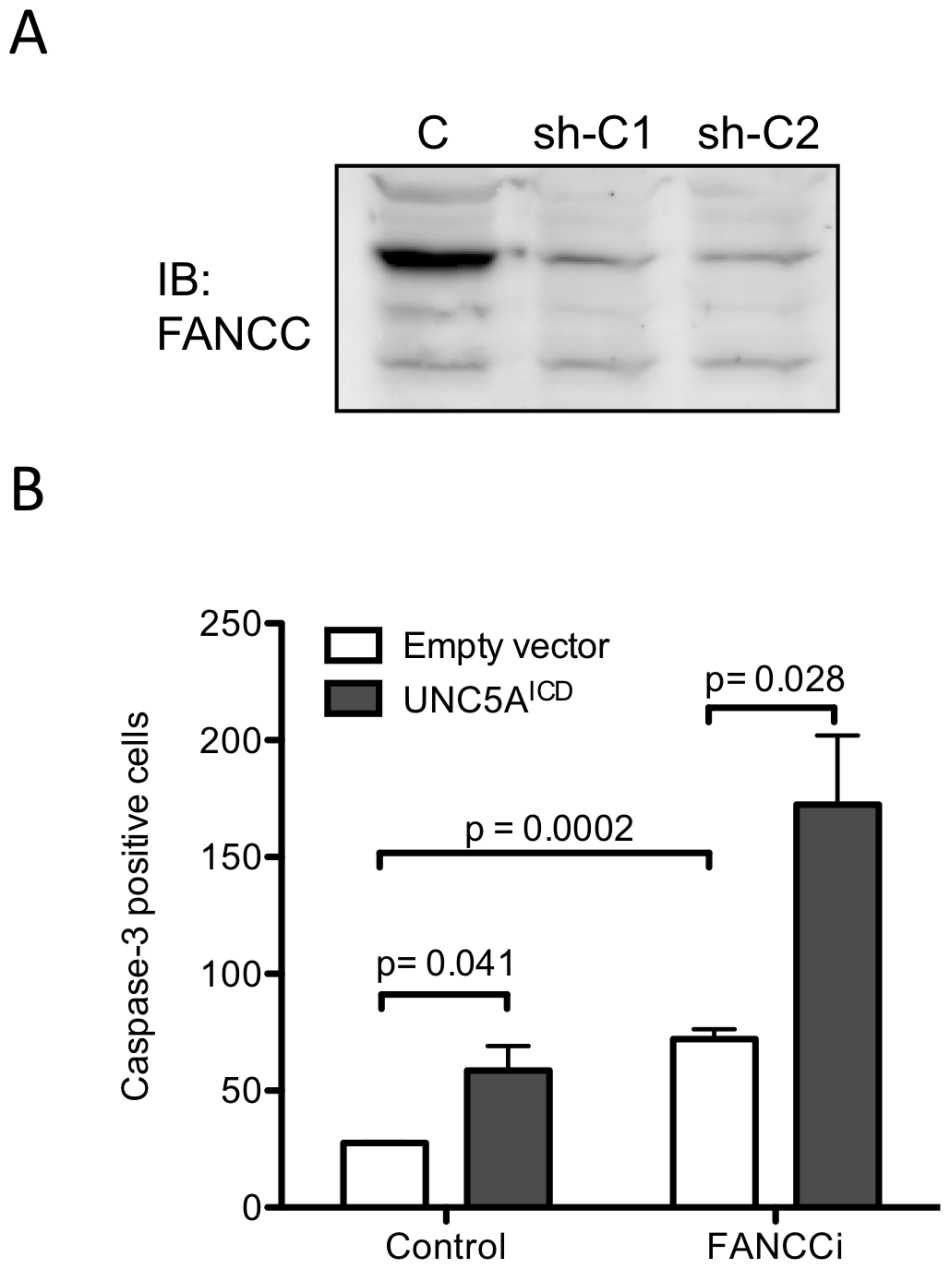
FANCC-depleted cells are sensitive to UNC5A-mediated apoptosis. SH-SY5Y cells were stably transduced with shRNA against FANCC (sh-C1 and sh-C2) or scrambled shRNA vectors (C). FANCCi and cells transduced with scrambled shRNA (control) were transfected with UNC5A^ICD^ or an empty vector. At 48 hours after transfection, cells were fixed and labeled with anti-cleaved-caspase-3 antibodies, and analyzed by fluorescence microscopy. Data are expressed as the mean cleaved-caspase-3 positive cells ± SEM from three separate experiments performed in duplicate. P values are indicated in the graph.

UNC5A has been shown to trigger apoptosis following caspase-dependent cleavage and release of its intracellular C-terminal domain (ICD) [30]–[32]. Because FANCC exhibits anti-apoptotic properties, is cleaved by a caspase and interacts with the intracellular domain of UNC5A [15], [33], we sought to determine the mechanistic impact of FANCC on UNC5A-mediated apoptosis. First, we measured apoptosis in cells over-expressing UNC5A with or without FANCC. HeLa cells were transfected with UNC5A^ICD^ and FANCC, and tested for caspase-3 activation. As expected, UNC5A^ICD^-over-expressing cells were prone to apoptosis and showed elevated numbers of caspase-3-positive cells whereas cells expressing FANCC with UNC5A^ICD^ exhibited reduced numbers of caspase-3-positive cells (Figure 5A–B). These results suggest that FANCC has a protective role in UNC5A-mediated apoptosis. Next, we evaluated the UNC5A-mediated apoptosis in cells expressing the FANCC protein harboring the L554P mutation found in patients with FA. Results show that the FANCC^L554P^ protein had a dominant negative effect over the endogenous wild-type FANCC and conferred no protective effect in cells expressing the UNC5A^ICD^ protein compared to the wild-type FANCC protein (Figure 5C). These results imply that cells with a defective FANCC protein would become more sensitive to UNC5A-mediated apoptosis. Consequently, we used FANCC-depleted cells to determine the impact of FANCC on UNC5A-mediated apoptosis. SH-SY5Y cells were transduced with lentiviral particles coding for shRNA against FANCC and subsequently, transfected with UNC5A^ICD^. Depletion of the FANCC protein was confirmed by Western blotting procedures (Figure 6A). Results showed that FANCC depletion as well as UNC5A^ICD^ overexpression increased apoptosis, as evidenced by the elevated number of caspase-3-positive cells (Figure 6B). In addition, expression of UNC5A^ICD^ together with FANCC depletion resulted in a dramatic increase in apoptosis. Together, these results suggest that FANCC modulates the cell sensitivity to UNC5A-mediated apoptosis.

## Discussion

**Figure 7:**
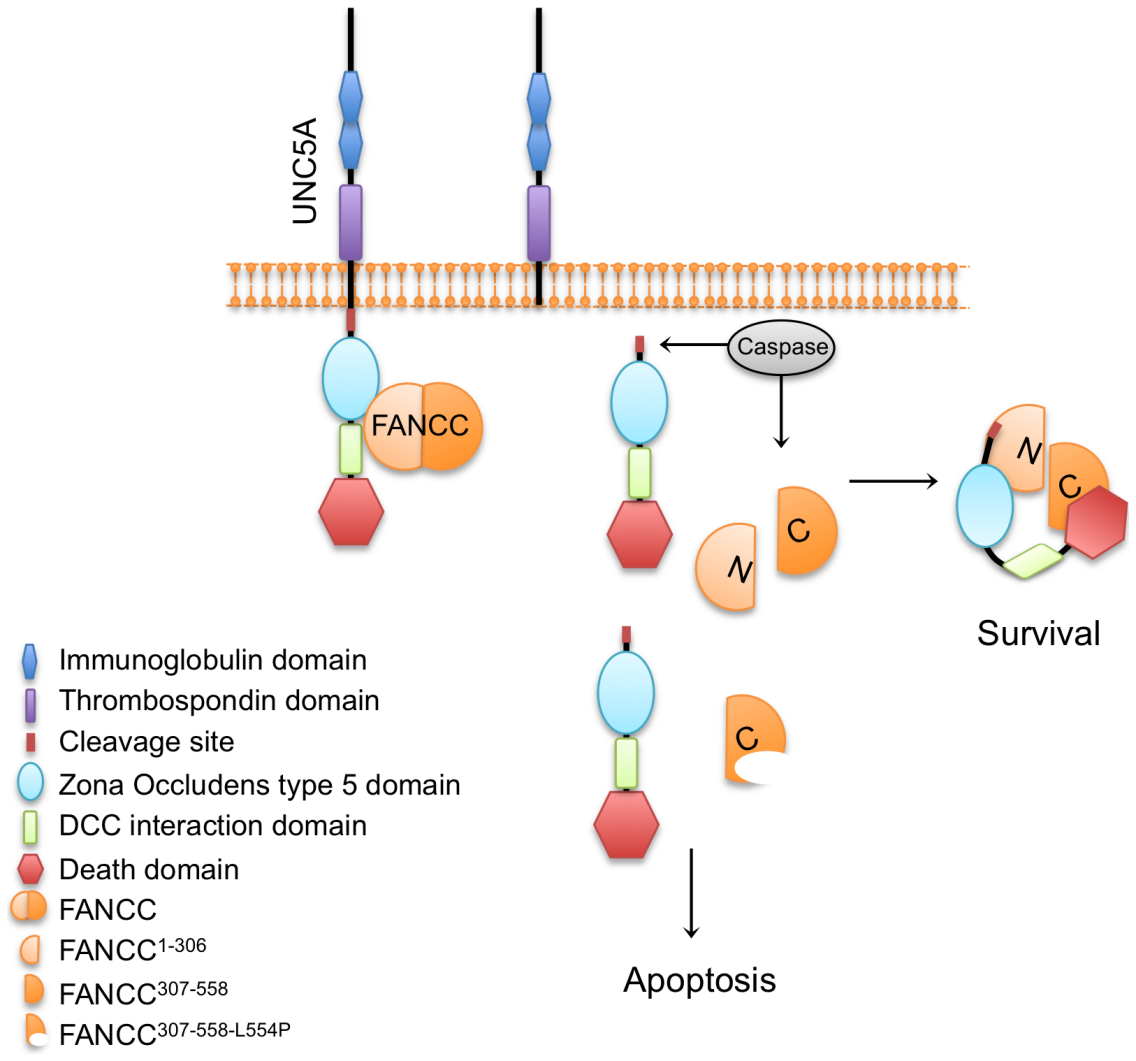
Schematic representation of FANCC interaction with UNC5A and possible role in apoptosis. Based on interactions studies, full length FANCC interacts with UNC5A via different parts of the intracellular domain. Upon apoptosis, FANCC and UNC5A are cleaved by a caspase. Cleaved FANCC fragments interact with UNC5A and interfere with the apoptosis signal (survival). In absence of FANCC or mutations in FANCC, UNC5A triggers apoptosis.

Our results provide the first evidence linking FANCC to UNC5A, a cellular receptor involved in tissue morphogenesis. Currently, very little is known about the role of FA proteins in developmental processes. FA proteins are thought to play a role in tissue homeostasis based on clinical disease phenotypes and developmental expression patterns [34]–[36]; however, a clear link has been elusive. Our data demonstrate that FANCC, which is also known as an anti-apoptotic protein, directly interacts with the pro-apoptotic dependence receptor UNC5A. Our results show that FANCC over-expression interferes with UNC5A-mediated apoptosis, whereas absence of a functional FANCC protein through depletion or mutation leads to increased UNC5A-mediated apoptosis. Thus, FANCC regulates the cell sensitivity to UNC5A-mediated apoptosis and may therefore act as a sensor of cellular stress and a switch between apoptosis and survival (Figure 7).

Based on expression studies and the role of UNC5A in apoptosis, it is considered to be a physiological regulator of tissue size and shape. For instance, UNC5 homologs are highly expressed in developing limbs [25]. Similarly, FA genes are expressed primarily in cells of mesenchymal origin that give rise to forelimb and hind limb tissues [34]–[36]. Consequently, defective FA genes result in limb malformations, and patients with FA can show absence or underdevelopment of thumbs and short or hypoplastic radii [3]. In view of our results that FANCC negatively impacts UNC5A's ability to induce apoptosis, we propose that dysregulation of UNC5A's apoptotic signal could lead to developmental defects similar to those observed in FA patients.

It is well known that FANCC suppresses apoptosis; FANCC-depleted cells or patient-derived cells with *FANCC* mutations show increased apoptosis induced by various cellular stressors, such as DNA damage, inhibitory cytokines, and oxygen radicals, whereas FANCC over-expression delays onset of apoptosis [1]. FANCC is also regulated by caspase-mediated cleavage, which inactivates its ability to suppress apoptosis [15]. The fact that FANCC directly interacts with the UNC5A C-terminal end harboring the DD suggests that interaction of both proteins may interfere with death signal transmission. It has been proposed that UNC5A mediates apoptosis via its C-terminal DD, although conflicting data suggest that the ZU-5 C-terminal domain, which binds the melanoma antigen gene D1 (MAGED1, formerly NRAGE), is required for apoptosis [23], [24]. The UNC5A homolog protein UNC5B was shown to directly interact with the death-associated protein kinase (DAPK) via its DD [37], [38]. This UNC5B/DAPK interaction was shown to be required for activation of the apoptotic cascade. Although UNC5A did not directly bind DAPK, regulation of DAPK activation via protein phosphatase 2A (PP2A) was shown to be required for UNC5A-mediated apoptosis [37], [38]. We identified a direct interaction between FANCC and the C-terminal region of UNC5A, including the ZU-5 and DD, suggesting that FANCC binding with UNC5A may interfere with UNC5A binding to apoptosis-promoting factors, such as MAGED1. Consequently, FANCC may act as a cellular sensor of UNC5A-mediated apoptotic cues and prevent or delay apoptosis depending on the tissue or cellular context.
